# The impact of sedation strategy on catheter stability in radiofrequency ablation for atrial fibrillation

**DOI:** 10.1016/j.hroo.2025.06.028

**Published:** 2025-07-08

**Authors:** Luke Byrne, Liam Maher, Caleb Powell, Mohamad Helmi, Gill Crowe, William Costello, Fiachra Clifford, Barry Kelly, Katie A. Walsh

**Affiliations:** 1 Department of Cardiology, Cork University Hospital, Wilton, Cork, Ireland; 2 School of Medicine, Trinity College Dublin, Dublin, Ireland; 3 Department of Anaesthetics, Cork University Hospital, Wilton, Cork, Ireland

**Keywords:** Atrial fibrillation, Radiofrequency ablation, Catheter stability, XYZ coordinates, Sedation strategy, General anesthesia, High-frequency jet ventilation, Conscious sedation

## Abstract

**Background:**

General anesthesia (GA) in radiofrequency ablation (RFA) for atrial fibrillation (AF) increases single procedure success rates and shortens procedure times vs conscious sedation (CS). In addition, high-frequency jet ventilation (HFJV) is associated with lower AF recurrence rates than conventional ventilation. Little data exist regarding the impact of sedation strategy on objective catheter stability and how this affects outcomes.

**Objective:**

This study aimed to (1) measure catheter stability using the standard deviation (SD) of XYZ coordinates of catheter location, obtained in patients undergoing RFA for AF, and (2) compare catheter stability in CS, GA, and GA + HFJV groups.

**Methods:**

All patients who underwent AF RFA at our center from April 2023 to June 2024 were eligible for inclusion in the study. Catheter stability was assessed using XYZ coordinates of catheter location, obtained via the CARTO 3 VisiTag module. The median SD of XYZ coordinates per ablation lesion was used to determine catheter stability.

**Results:**

A total of 28 patients were included in the study, 8 in the CS group, 10 in the GA group, and 10 in the GA + HFJV group; 1,979,105 XYZ coordinates of RFA catheter location were analyzed. GA demonstrated an improvement in catheter stability compared with CS (median [interquartile range]) (0.54 [0.3–0.89] vs 1.51 [0.95–2.3], *P* < .001). GA + HFJV demonstrated further improvement in catheter stability vs GA (0.47 [0.25–0.87], *P* = .017).

**Conclusion:**

The SD of XYZ coordinates of catheter location, obtained via the CARTO 3 VisiTag module, can be used to assess catheter stability during AF RFA. GA + HFJV offers superior catheter stability compared with GA and CS.

## Graphical abstract

**Figure:**
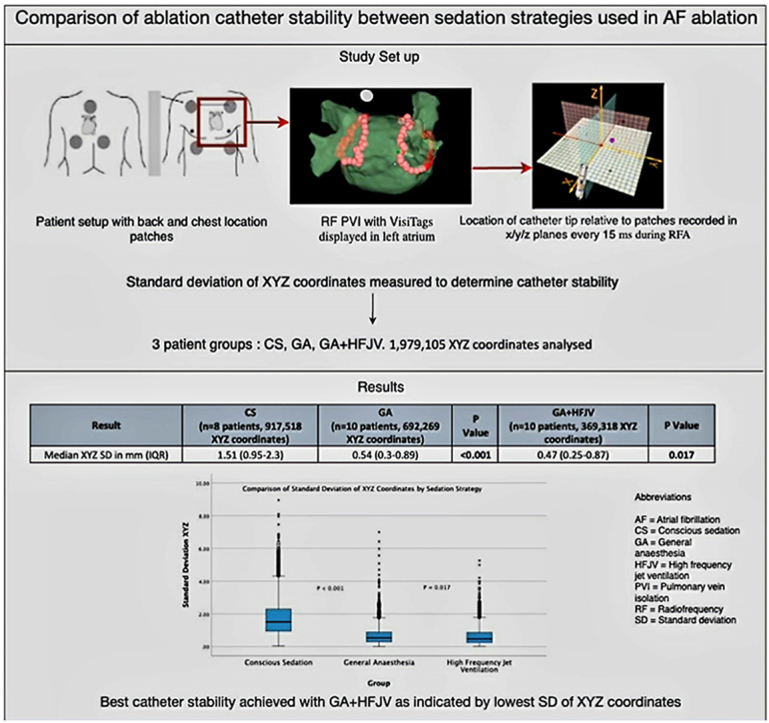

**Key Findings**

▪ Objective catheter stability can be measured using the standard deviation (SD) of XYZ coordinates obtained from the CARTO VisiTag module, with lower SD representing improved stability.
▪ The use of general anesthesia resulted in a significant improvement in catheter stability over conscious sedation during radiofrequency ablation (RFA) for atrial fibrillation (AF).
▪ Improved catheter stability was seen in the high-frequency jet ventilation group vs the conventional mechanical ventilation group during AF RFA.

## Introduction

The use of catheter ablation (CA) for the treatment of atrial fibrillation (AF) has increased and is now recommended as a first-line treatment option, within a shared decision rhythm control strategy, in patients with paroxysmal AF.^1^ This increase has been driven by reduced rates of AF recurrence and reduced AF burden after CA, with improved patient-reported quality of life compared with antiarrhythmic drug therapy.^2^ CA has also been associated with a reduction in mortality and hospitalizations for patients with AF and heart failure.^3^^,^^4^ The use of general anesthesia (GA) has become the preferred method of sedation among electrophysiologists for radiofrequency (RF) ablation (RFA) of AF. A writing group survey performed by Calkins et al^5^ reported that 73% used this approach, 14% used moderate conscious sedation (CS), and 8% used high-frequency jet ventilation (HFJV). In this report, a lack of anesthesiology availability was cited as the major reason for not implementing GA. The use of GA during RFA provides better patient comfort, less patient movement, and control of tidal volumes. This allows for less respiratory variation, less shift in the electroanatomic map (EAM), and, in theory, better catheter stability.^6^ The use of GA has been shown to increase the rate of single procedure success and shorten procedural and fluoroscopy times. In addition, better patient satisfaction has been reported with this method.^7^^,^^8^

The use of HFJV implements a low-tidal-volume, high-frequency ventilation strategy, providing less respiratory motion and diaphragmatic movement.^9^ Shorter procedure times, fewer pulmonary vein (PV) reconnections, and greater freedom from AF have been reported with the use of HFJV.^10^^,^^11^ Furthermore, improved catheter stability vs standard GA, measured via contact force (CF) standard deviation (SD), has been reported.^12^ Whether the use of HFJV provides further incremental improvement in catheter stability compared with GA and conventional ventilation is unknown.

This study aimed to:

(1) Measure catheter stability using the SD of XYZ coordinates of RFA catheter location obtained using the CARTO 3 VisiTag module in patients undergoing AF RFA
(2) Determine the impact of different sedation strategies (CS, GA, and GA + HFJV) on RFA catheter stability in patients undergoing AF RFA

## Methods

### Patient selection

All patients who underwent AF RFA at our center from April 2023 to June 2024 were screened for eligibility. Both index and redo cases were assessed. Only cases in which point-by-point RFA using the CARTO 3 VisiTag module was used were included in the study. Baseline demographics and clinical details were accessed from electronic medical records. Procedural details, including procedure time, ablation time, ablation lesion count, first pass isolation (FPI), fluoroscopy time, and fluoroscopy dose, were obtained from electronic procedural reports.

### AF ablation workflow

#### Ablation procedure

An 8F Agilis deflectable sheath (Abbott Laboratories, Chicago, IL) was introduced into the right atrium, and using a Brockenbrough needle (BRK-1 XS, Abbott Laboratories) and intracardiac echo guidance, a single transeptal puncture was performed. Intravenous (IV) heparinized saline was infused through the transeptal sheath. A 3-dimensional (3D) EAM of the left atrium was obtained using a Pentaray catheter (Biosense Webster, Diamond Bar, CA). Point-by-point RFA was performed using an open-irrigated, CF-sensing RFA catheter (ThermoCool SmartTouch, Biosense Webster) or QDOT Micro catheter (Biosense Webster). Wide antral circumferential ablation was performed using 30–40 W of RF energy to achieve PV isolation (PVI). Ablation index (AI) was used with a target AI of 500 on the anterior wall and 350 on the posterior wall. An interlesion distance of ≤5 mm was targeted. PVI was confirmed with demonstration of entry and exit block during pacing at 20 mA with a pulse width 2 ms in each of the PVs. FPI was defined as the entrance and exit block that was achieved on the first attempt via wide antral circumferential ablation of the PVs. When posterior wall isolation was required, floor and roof lines were performed, and supplemental ablation was performed within the posterior wall if required. Posterior wall isolation was confirmed by demonstrating entry and exit block in the posterior wall during pacing at 20 mA with a pulse width of 2 ms. Pacing during the procedure was only implemented in the context of bradycardia.

#### VisiTag settings

Respiratory gating was performed at the outset of the procedure. To receive a VisiTag, the catheter must not be moved more than 3 mm for a minimum of 3 seconds. A CF stability filter was also applied, in which CF must be greater than 3 g for 25% of the ablation session, and the tag size was set to 3 mm.

#### QDOT catheter

The QDOT Micro catheter is a CF-sensing, open-irrigated RF catheter with the capability to deliver very-high-power short-duration (VHPSD) RF energy at a power of 70–90 W. High-power energy delivery results in shorter ablation times, which may affect overall catheter stability. Our center began using the QDOT catheter later in the study period, resulting in use only in patients allocated to the HFJV group. To mitigate any potential effect of VHPSD on catheter stability, only lesions created using standard power settings of 30–40 W were included for this analysis.

### Sedation strategies

#### CS

Patients undergoing CS were administered 2 mg of IV midazolam and 50 μG of IV fentanyl at the beginning of the procedure. Their vital signs and comfort levels were observed throughout the procedure by noninvasive means. Further boluses of 1 mg IV midazolam and 25 μG IV fentanyl were administered as needed to optimize the patient’s comfort level throughout the procedure.

#### GA

All patients in the GA group were managed by a consultant anesthetist. Prior to induction, standard monitoring, including noninvasive blood pressure, continuous electrocardiogram, pulse oximetry, and Entropy monitoring (GE HealthCare, Chicago, IL), was applied. Patients were intubated orotracheally after IV induction with a short-acting opioid and propofol with or without rocuronium at the discretion of the treating anesthesiologist. Anesthesia was maintained using target-controlled infusions of propofol and remifentanil. Boluses of rocuronium were administered as required for continuous neuromuscular blockade.

#### Conventional mechanical ventilation

Patients in the CMV group, or the standard GA group, were ventilated using a pressure control mode with volume guarantee via the Aisys (GE HealthCare) anesthetic machine ventilator. A positive end-expiratory pressure of 5–8 cm H_2_O was used in all patients. Tidal volumes of 300–350 mL were targeted, and the respiratory rate was adjusted to maintain an end-tidal carbon dioxide of 4.5–5.5 kPa. The fraction of inspired oxygen was adjusted to maintain oxygen saturations of >96%.

#### HFJV

Patients in the GA + HFJV group were initially managed with conventional ventilation and moved to HFJV using the Monsoon 4 device (Acutronic) prior to the beginning of the procedure. Jet ventilation was delivered via a swivel adaptor with a standard Luer-lock connector connected directly into the circle breathing circuit at the endotracheal tube. Standard settings were as follows: fraction of inspired oxygen of 0.45–0.5, cycle frequency of 100/min, inspiratory time of 40%, driving pressure of 2–2.5 bar, and mean airway pressure of 20–25 mbar. A venous blood gas was taken at baseline and 15 and 30 minutes after starting jet ventilation, then every 30 minutes thereafter. Settings were titrated to maintain a partial pressure of venous carbon dioxide of <7 kPa and oxygen saturation of >96%.

### Assessment of catheter stability

#### XYZ coordinates

The CARTO 3 VisiTag module obtains a series of XYZ coordinates of RFA catheter location every 15 ms during RFA delivery, resulting in a large volume of data for each ablation lesion. This provides information on catheter tip position in 3 orthogonal planes within the left atrium, providing a 3D reference frame. This is achieved via the CARTO 3 Body Coordinate System, obtained using the relative positions of 3 patches on the patient’s back, which are connected to each other and to a location pad. Each set of coordinates provides the distance of the catheter tip from the back patches, measured in millimeters. Raw catheter position data, in the form of XYZ coordinates, were exported to SPSS for statistical analysis (IBM Corp, Armonk, NY). To measure variation in catheter movement, the SD of coordinates on the X, Y, and Z axes was calculated for each ablation lesion. Given that the SD is invariant to the difference in reference point between patients, these values were compiled and grouped as per sedation strategy. The SDs of X, Y, and Z coordinates in each group (CS, GA, and GA + HFJV) were then compared. A lower SD value represents less variation in catheter movement and better stability.

The following formulas were used to calculate the SD of X, Y, and Z coordinates, respectively, for each ablation lesion:

$$SDx=\sqrt{\frac{1}{N-1}\sum_{i=1}^{N}{\left(X_{1}-\bar{X}\right)}^{2}}$$

$$SDy=\sqrt{\frac{1}{N-1}\sum_{i=1}^{N}{\left(Y_{1}-\bar{Y}\right)}^{2}}$$

$$SDz=\sqrt{\frac{1}{N-1}\sum_{i=1}^{N}{\left(Z_{1}-\bar{Z}\right)}^{2}}$$

#### Statistical analysis

Categorical variables are presented as a number (percentage) ± SD. Fisher’s exact test and χ^2^ test were used to determine statistical significance. Continuous variables are presented as mean ± SD and were compared for significance using the Student *t* test where the data are normally distributed. In cases where data are abnormally distributed, it is presented as median (interquartile range [IQR]) and the Mann-Whitney U test was used for statistical analysis. Datasets were analyzed for skewness and kurtosis to determine whether nonparametric analysis was required. A 2-tailed *P* < .05 was used to determine statistical significance. Statistical analysis was performed using SPSS software.

#### Ethical statement

This study was conducted in line with the Declaration of Helsinki and was approved by a local ethics committee under the reference number ECM 4 (o) 10/09/2024.

#### Consent statement

An informed consent was not obtained given that all data were retrospectively collected and anonymized.

## Results

In our institution, the routine use of GA for AF RFA was introduced in July 2023. Prior to this date, all AF RFA procedures were performed under CS. From February 2024, HFJV became available in our center and was subsequently used for AF RFA.

A total of 28 patients met the inclusion criteria during the study period: 8 in the CS group, 10 in the GA group, and 10 in the GA + HFJV group. A total number of 1754 VisiTag lesions, and 1,979,105 XYZ coordinates were analyzed across all patients. The average (±SD) number of lesions and median (IQR) XYZ coordinates per patient, across all groups, were 63 (±41) and 61,500 (36,811–96,707), respectively.

A median follow-up of 8.9 months was obtained. All groups were similar in terms of age, gender, ablation indication, AF subtype, and the rate of index vs redo procedures. The number of veins isolated and the rate of posterior wall isolation were also similar among the groups. All patients in the CS and GA groups underwent ablation with a SmartTouch catheter vs 3 patients (30%) in the GA + HFJV group (*P* = .002). The remaining patients in the GA + HFJV group (70%) underwent ablation with a QDOT catheter. A summary of patient demographics is presented in Table 1.

**Table 1:** Baseline demographics and procedural parameters

Demographic	CS (n = 8)	GA (n = 10)	*P* value	GA + HFJV (n = 10)	*P* value
Men (%)	6 (75)	8 (80)	.79	8 (80)	.69
Mean age	60.9	55.2	.31	62.7	.17
Paroxysmal AF (%)	3 (37.5)	5 (50)	.59	5 (50)	1
Persistent AF (%)	5 (62.5)	5 (50)	.59	5 (50)	1
Redo procedure (%)	1 (12.5)	3 (30)	.37	2 (20)	.6
Procedural parameter					
Mean procedure time (min) (±SD)	100.9 (±33)	72.9 (±17)	.04∗	75.5 (±16.8)	.73
Mean ablation time (s) (±SD)	1949.9 (±831)	1174.2 (±428)	.03∗	750 (±396)	.07
Mean fluoroscopy time (s) (±SD)	267.7 (±129)	220.6 (±52.5)	.33	198.9 (±82.3)	.48
Mean radiation dose (cGy/cm^2^) (±SD)	368.1 (±191.4)	289.9 (±110.1)	.32	248.33 (±87.8)	.44
Mean number of veins attempted	3.6	3	.38	3.8	.38
Posterior wall isolation (%)	2 (25)	4 (40)	.5	6 (60)	.37
First pass isolation (%)	5 (62.5)	8 (80)	.41	9 (90)	.53
Complications	0	0	n/a	0	n/a
SmartTouch (%)	8 (100)	10 (100)	n/a	3 (30)	.002∗
QDOT (%)	0	0	n/a	7 (70)	.002∗

This table outlines baseline demographics and procedural parameters. Data are presented as a percentage or mean ± SD where appropriate. *P* values are presented as comparisons between the GA and CS groups and between the GA + HFJV and GA groups, respectively.

AF = atrial fibrillation; CS = conscious sedation; GA = general anesthesia; HFJV = high-frequency jet ventilation; SD = standard deviation; n/a = not available.

∗ Statistically significant.

### Procedural results

Procedure time was significantly shorter in the GA group than the CS group (72.9 ± 17 minutes vs 100.9 ± 33 minutes, *P* = .04). There was no difference in procedure time between the GA and GA + HFJV groups (75.5 ± 16.8 minutes, *P* = .73). Ablation time was also significantly lower in the GA group vs the CS group (1174.2 ± 428 seconds vs 1949.9 ± 831 seconds, *P* = .03). There was no significant difference in ablation time in the GA + HFJV group compared with GA (750 ± 396 seconds, *P* = .07). No difference was seen across the groups in terms of fluoroscopy time and radiation dose (267 ± 129 seconds, 220.6 ± 52.5 seconds, and 198.9 ± 82.3 seconds, *P* = .33 and *P* = .48, respectively, and 368 ± 191.4 cGy/cm^2^, 289.9 ± 110.1 cGy/cm^2^, and 248.33 ± 87.8 cGy/cm^2^, *P* = .32 and *P* = .44, for CS, GA, and GA + HFJV, respectively). No difference was noted among the groups in terms of FPI, with FPI achieved in 62.5% of the CS group, 80% of the GA group (*P* = .41), and 90% of the GA + HFJV group (*P* = .53). Finally, no procedural complications occurred in any patients during the study period.

### Catheter stability

The median (IQR) number of coordinates per patient was 114,323 (84,046–159,389) in the CS group. This was significantly lower in both the GA group (69,611 [50,327–85,130], *P* = .02) and the GA + HFJV group (41,751 [14,453–54,366], *P* < .001). The number of coordinates recorded in the GA + HFJV group was also significantly lower than that recorded in the GA group (*P* = .01).

There were significantly fewer VisiTags (mean ± SD) created per procedure in the GA group than the CS group (47 ± 19 vs 110 ± 40, *P* < .001). No difference was observed in the number of VisiTags created in GA + HFJV patients compared with standard GA (40 ± 24, *P* = .44).

**Figure 1:**
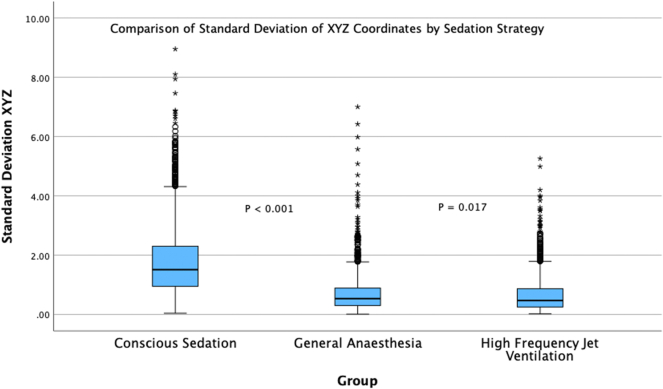
Comparison of the standard deviation of XYZ coordinates by sedation strategy. A boxplot representation of the median standard deviation of XYZ coordinates (interquartile range) measured in millimeters (mm) across the 3 groups, conscious sedation, general anesthesia, and general anesthesia + high-frequency jet ventilation. The Mann-Whitney U test was used to determine statistical significance among the groups owing to an unequal distribution of data across the groups, with rightward skew evident.

An incremental improvement in catheter stability was demonstrated across the 3 subgroups as measured by the SD of XYZ coordinates. The median SD of XYZ coordinates seen in the CS group was 1.51 mm (0.95–2.3). A significantly lower value was noted in the GA group at 0.54 mm (0.3–0.89) (*P* < .001), representing a 64% relative improvement in catheter stability over CS. Catheter stability was also significantly better in the GA + HFJV group than the CS group at 0.47 mm (0.25–0.87) (*P* < .001). Furthermore, a 13% relative improvement compared with GA was seen in the GA + HFJV group (*P* = .017). The differences in terms of catheter stability among the 3 groups are presented in Figure 1 and Table 2.

**Table 2:** Results

Result	CS(n = 8 patients, 917,518 XYZ coordinates)	GA(n = 10 patients, 692,269 XYZ coordinates)	*P* value	GA + HFJV(n = 10 patients, 369,318 XYZ coordinates)	*P* value
Median XYZ SD in mm (IQR)	1.51 (0.95–2.3)	0.54 (0.3–0.89)	<.001	0.47 (0.25–0.87)	.017
Median number of XYZ coordinates (IQR)	114,323 (84,046–159,389)	69,611 (50,327–85,130)	.02	41,751 (14,453–54,366)	.01
Mean number of VisiTags (±SD)	110 (±40)	47 (±19)	<.001	40 (±24)	.44

This table demonstrates the difference in XYZ parameters among the 3 groups. Data are presented as median (interquartile range) or mean (±SD) where appropriate. *P* values are presented comparing the GA vs CS groups and HFJV vs GA groups, respectively.

CS = conscious sedation; GA = general anesthesia; HFJV = high-frequency jet ventilation; IQR = interquartile range; SD = standard deviation.

**Figure 2:**
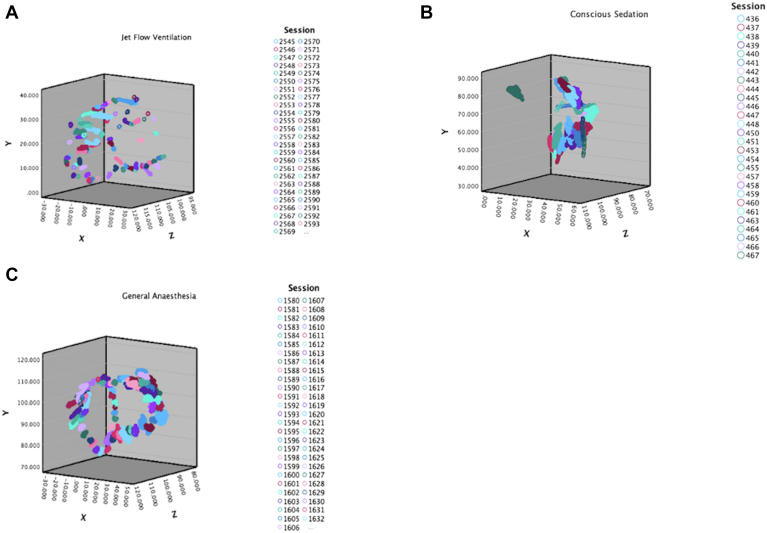
Boxplot comparison of XYZ coordinates in patients with the best stability in each group. A 3-dimensional scatter plot of XYZ coordinates from patients with the best catheter stability in each of the groups. Each VisiTag lesion is color coded and plotted on 3 orthogonal axes. The surface area of each VisiTag represents the variability in catheter movement during ablation at that site. A decrement in lesion surface area is well demonstrated on this scatter plot, with the general anesthesia + high-frequency jet ventilation set showing the best radiofrequency ablation catheter stability.

The patients from each group with the best catheter stability were identified, and their XYZ coordinates are represented on a 3D scatter plot in Figure 2. This illustrates the degree of catheter movement among the 3 individual patients in each group. The median SD of XYZ coordinates in these cases was 1.02 in the CS group, 0.42 in the GA group, and 0.21 in the HFJV group.

**Figure 3:**
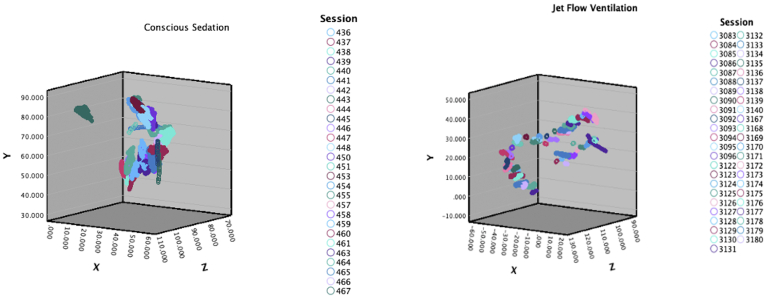
Boxplot comparison of XYZ coordinates in the same patient. A 3-dimensional scatterplot of XYZ coordinates taken from the same patient who underwent their initial PVI under conscious sedation. Their second procedure was performed at a later stage with general anesthesia + high-frequency jet ventilation. A clear improvement in radiofrequency ablation catheter stability is demonstrated in the same patient with the use of general anesthesia + high-frequency jet ventilation. PVI = pulmonary vein isolation.

One patient underwent their index PVI under CS (Figure 2B) and had a redo procedure using GA + HFJV owing to AF recurrence. A 3D scatterplot comparing catheter position for both procedures is outlined in Figure 3.

## Discussion

### Main findings

The main findings of this study are :

(1) The SD of XYZ coordinates obtained from the CARTO 3 VisiTag module can be used to assess catheter stability, with a lower SD representing less catheter movement during RFA delivery.
(2) The mode of anesthesia implemented during AF RFA affects catheter stability.

In this cohort of patients, which included analysis of 1,979,105 XYZ coordinates of RFA catheter location, both the GA and GA + HFJV groups had a significantly improved median SD of XYZ coordinates compared with the CS group, demonstrating a 64% and 69% relative improvement in catheter stability, respectively. In addition, a significant relative improvement in catheter stability of 13% was also seen in the GA + HFJV group compared with the GA group, suggesting that this sedation strategy confers the most stability.

Significantly fewer XYZ coordinates were created across the 3 groups, with the lowest number of coordinates seen in the GA + HFJV group. This difference is presumably observed as the target AI is reached more quickly with better catheter stability. The shorter the duration of ablation per lesion, the fewer the XYZ coordinates recorded by the system.

Significantly more VisiTags were created in the CS group than the GA and GA + HFJV groups. This is explained by less catheter stability during RFA delivery under CS. When the catheter moves during RFA, the operator turns off ablation, repositions the catheter back at the desired location, and then restarts RFA. This results in the delivery of more lesions (but with fewer of them reaching the target AI).

To the best of our knowledge, this is the first study to use the SD of XYZ coordinates, obtained via the VisiTag module on the CARTO 3 system, to measure catheter stability in patients undergoing AF RFA. Furthermore, our study is the first to report an incremental improvement in catheter stability across 3 sedation strategies implemented for AF RFA.

### Current evidence on the use of GA and HFJV in AF RFA

Improvements in AF outcomes with the use of GA have been reported in previous trials; however, little data exist regarding the impact of catheter stability on these outcomes. A recent large-scale cohort study found a 26% higher risk of AF recurrence with the use of CS than GA.^13^ This finding has been echoed in other studies with Di Biase et al^8^ demonstrating fewer rates of PV reconnection associated with the use of GA. Improved CF parameters, RF duration time, force-time integrals, and reduced gap formation have also been reported with the implementation of GA.^14^ The significant improvement in catheter stability demonstrated in our paper suggests that this plays an important role in improved outcomes associated with the use of GA compared with CS.

Hutchinson et al^11^ were the first to report on the benefit of HFJV in reducing the risk of AF recurrence by demonstrating that the use of all 3 components of HFJV, EAM, and steerable introducer sheaths reduced the recurrence rate by 12%.Click or tap here to enter text. These outcomes were reproduced in a study by Sivasambu et al^15^ in which both mean CF and maintenance of CF of >5 g were significantly improved with the use of HFJV, suggesting improved lesion quality with this method. In this paper, HFJV was associated with an increased use of vasopressors periprocedurally; however, no significant safety concerns were reported. Furthermore, Osorio et al^16^ reported that the use of HFJV resulted in an absolute reduction in atrial arrhythmia recurrence by 6.3% compared with standard ventilation.Click or tap here to enter text.

### SD of XYZ coordinates: Rationale for use in assessing catheter stability

Using the SD of XYZ coordinates, taken every 15 ms during RFA delivery, generates a large amount of data on catheter location during each ablation lesion. Despite the small number of patients included in this study, a large volume of data were analyzed, amounting to almost 2 million XYZ coordinates across the 3 groups. This ensured that our study was powered to determine whether a significant difference in catheter stability exists. The use of XYZ coordinate data to assess stability may confer advantages over previously reported methods such as CF SD, which has been reported in previous trials.^12^ CF is made up of 2 components: the magnitude of surface area of the electrode in direct contact with the tissue and catheter stability.^17^ Although CF variability may be an indirect measure of catheter stability, it is also affected by the degree of force applied to the catheter. In addition, stable CF may be seen in the context of poor catheter stability, such as catheter sliding along the ridge between the left atrium and the left atrial appendage. CF measurement accuracy is also affected by catheter vector orientation, with lower accuracy reported during parallel vs perpendicular tissue-catheter contact.^18^ The calculation of the SD of XYZ coordinates for a given VisiTag provides data on the degree of variability of catheter tip movement irrespective of force applied. A similar method was described by Kuno et al^19^ who examined the difference in catheter stability in patients undergoing PVI with GA vs CS. Their study examined the total distance traveled by the catheter as a marker of stability, using the EnSite Precision mapping system.Click or tap here to enter text. Our study describes similar findings to this trial, suggesting the validity of our method.

Overall, the findings of our paper suggest that some of the procedural benefits seen with the application of both GA and GA + HFJV may be secondary to the incremental improvement in catheter stability offered by these methods.

### Limitations

The small sample size of 28 patients leaves the study underpowered to determine whether a significant difference in clinical outcomes and freedom from AF exists among the groups. It should be noted that 7 patients in the GA + HFJV group underwent ablation with a QDOT catheter. To overcome this, only lesions created using a power setting of 30–40 W were included in the analysis. Given the similarities in catheter topography, this is unlikely to have a significant impact on the validity of our results. Whether the use of VHPSD ablation techniques confers an objective difference in catheter stability over standard energy delivery requires additional study. Furthermore, our paper did not report on the periprocedural incidence of vasopressor requirement with the use of HFJV, which has previously been associated with HFJV use.^16^ However, no significant procedural complications were noted in our cohort. Finally, our study took place in 1 center and with all AF RFA performed by 1 operator, which may limit the generalization of our findings to other centers and operators.

## Conclusion

The SD of XYZ coordinates of the RFA catheter location can be analyzed to assess catheter stability. An incremental improvement in catheter stability, assessed using the SD of XYZ coordinates, was seen across the different sedation strategies offered for AF RFA. The best catheter stability was achieved using GA + HFJV. Improvement in catheter stability across the 3 groups may account at least in part for the improved procedural outcomes seen with the use of GA and GA + HFJV when implemented for AF RFA. Further research in this area may establish whether the results of this study are reproducible in other centers/operators. Whether catheter stability, as measured using the SD of XYZ coordinates, predicts AF recurrence rates or AF burden requires prospective investigation.
